# Selecting and describing control conditions in mobile health randomized controlled trials: a proposed typology

**DOI:** 10.1038/s41746-023-00923-7

**Published:** 2023-09-30

**Authors:** Simon B. Goldberg, Shufang Sun, Per Carlbring, John Torous

**Affiliations:** 1 Department of Counseling Psychology, University of Wisconsin–Madison, Madison, WI USA; 2 Center for Healthy Minds, University of Wisconsin–Madison, Madison, WI USA; 3 Department of Behavioral and Social Sciences, Brown University School of Public Health, Providence, RI USA; 4 International Health Institute, Brown University School of Public Health, Providence, RI USA; 5 Mindfulness Center, Brown University School of Public Health, Providence, RI USA; 6 Department of Psychology, Stockholm University, Stockholm, Sweden; 7 Department of Psychiatry, Beth Israel Deaconess Medical Center, Harvard Medical School, Boston, MA USA; 8 Harvard Business School, Boston, MA USA

**Keywords:** Randomized controlled trials, Outcomes research, Health policy

## Abstract

Hundreds of randomized controlled trials (RCTs) have tested the efficacy of mobile health (mHealth) tools for a wide range of mental and behavioral health outcomes. These RCTs have used a variety of control condition types which dramatically influence the scientific inferences that can be drawn from a given study. Unfortunately, nomenclature across mHealth RCTs is inconsistent and meta-analyses commonly combine control conditions that differ in potentially important ways. We propose a typology of control condition types in mHealth RCTs. We define 11 control condition types, discuss key dimensions on which they differ, provide a decision tree for selecting and identifying types, and describe the scientific inferences each comparison allows. We propose a five-tier comparison strength gradation along with four simplified categorization schemes. Lastly, we discuss unresolved definitional, ethical, and meta-analytic issues related to the categorization of control conditions in mHealth RCTs.

## Introduction

The World Health Organization defines mobile health (mHealth) as the “use of information and communication technologies in support of health and health-related fields”^1^. There has been immense research interest in this area in the past decade. For example, a recent review of mobile phone-based interventions for mental health included 145 randomized controlled trials (RCTs) and 47,940 participants, with most RCTs published in the last 10 years^2^. Many mHealth tools are being tested and, beginning in 2020, some have earned Food and Drug Administration (FDA) approval^3,4^. mHealth is projected to become a USD$250 billion industry by 2026^5^.

RCTs are central for establishing efficacy in medicine, with the double-blind placebo-controlled RCT long being the gold standard^6,7^. The ability to blind patient and provider to group assignment allows a rigorous test of efficacy that accounts for non-specific factors such as expectancy. This method is ideal for testing pharmacological interventions where masking can be relatively easily done through placebo medications.

Double-blind placebo-controlled trials have also been used to test non-pharmacological interventions, such as surgery^8^. However, the translation of this design to behavioral interventions is not straightforward. It can be difficult to standardize interventions that include human interaction, as in psychotherapy^9^. Moreover, behavioral interventions commonly include multiple components that are not easily separated. Although mHealth interventions may or may not include human interaction, these approaches often include a combination of features (e.g., mood tracking, mindfulness)^10^ which makes isolating active ingredients difficult without close attention to control conditions.

Several guidelines have been published for selecting and describing control conditions in behavioral interventions^11–14^. These guidelines describe a variety of control condition types and factors to be considered when selecting a control group (e.g., trial phase and aim, need to balance internal validity and statistical power)^11–13^. However, there are no established guidelines for designing and categorizing control conditions for mHealth RCTs^15,16^ (although some meta-analyses of the mHealth literature have tested this design feature as a moderator)^17^. Extant guidelines for behavioral interventions do not necessarily map neatly onto the mHealth literature where intervention and control conditions vary widely in form, intensity, and delivery setting. Currently, the same term—“control”—can be used across RCTs to refer to quite different control conditions^18^. “Treatment-as-usual” (TAU), “care-as-usual” and “usual care” are notoriously ambiguous designations. For example, TAU can be quite minimal (e.g., permission to pursue treatment outside of the study, referrals to non-study-related providers)^19^ or fairly extensive (e.g., receipt of pharmacotherapy and/or psychotherapy)^20–22^. At times, studies may also refer to a group receiving TAU as a waitlist control^23^. Ambiguity defining and categorizing control conditions impacts attempts to synthesize trends across mHealth RCTs. Meta-analyses commonly combine substantially different control conditions, which complicates interpretation of results^24^.

As mHealth moves toward wider adoption within health systems^3^, there is a need for a consistently applied typology of control conditions. A lack of clarity can lead to misunderstanding and disappointment^25^. If control conditions are interpreted as a homogeneous group, interventions compared with stronger control conditions may be perceived as less effective^18,26^. Conversely, it may become clear relatively late in the evaluation process that interventions with promising pilot data do not outperform even minimally active controls^27^. Clarifying the universe of mHealth control condition types can help highlight the scientific questions each type can reasonably address and comparison strength each type provides^12^.

## Current study

Building on guidelines developed for behavioral interventions^11–14^, we propose a typology for selecting and defining control condition types in the context of mHealth RCTs. We highlight relevant dimensions of differentiation and provide a decision tree for identifying control conditions. We intend this typology to be comprehensive and capable of differentiating between the wide variety of control conditions appearing in the mHealth literature. For this reason, we define 11 distinct types. We clarify what scientific question each can answer and propose a five-tier comparison strength grading scheme. In addition, we propose simplified categorizations (from five to two categories) which may be more usable for meta-analysis. Lastly, we highlight open questions and future directions for control conditions in mHealth RCTs.

## Proposed typology

### Relevant dimensions

Our proposed typology considers five dimensions (Table 1): intended to be therapeutic, intensity, intensity match, masking/expectancy match, and received by both groups. Therapeutic intention is a critical first consideration^28^. Control conditions that are not intended to be therapeutic have an upper bound on the comparison strength they provide. Studies with non-therapeutic control conditions are still scientifically valuable as they can establish the impact of non-specific factors^12^, but they cannot provide the highest comparison strength. Control condition intensity is an important second consideration, particularly in the mHealth context where many focal interventions are self-guided and of potentially modest intensity. A third consideration is whether the intensity is matched between the focal intervention and the control condition. This can be challenging to establish, particularly given inconsistencies in the definition and reporting of engagement with mHealth interventions^29,30^. A fourth consideration is whether the treatment condition is masked and in such a way that expectancy is likely to be matched between focal and control conditions. This is arguably the characteristic feature of an ideal placebo control and any control conditions capable of clarifying intervention effects beyond non-specific factors^31,32^. It is important to acknowledge that clinical trialists, despite their best intentions, may or may not succeed in matching expectancy, and the development of psychotherapy placebos remains a challenging (and some might say impossible)^9,33^ task. A fifth consideration is whether the control condition is received by both focal intervention and control groups. This is especially relevant in the case of TAU controls in which both focal intervention and control conditions receive the TAU^23^.

**Table 1:** Eleven-category scheme with relevant dimensions of differentiation between focal intervention and control condition.

Control condition	Example	Intended to be therapeutic (yes/no)	Intensity (none/low/high)	Intensity match (no/maybe/yes)	Masking/expectancy match (no/maybe/yes)	Received by both groups (no/maybe/yes)
No Treatment	Waitlist vs. virtual reality smartphone app^61^	No	None	No	No	No
Placebo-Minimal	List-making app vs. meditation app^62^	No	Low	No	Maybe	No/maybe
Placebo-Active	Attention training app vs. attentional bias modification app^63^	No	High	Yes	Yes	No
TAU-Minimal	Antiretroviral psychoeducation + mood monitoring texts vs. antiretroviral psychoeducation + mood monitoring texts + personalized adherence reminder texts^64^	Yes	Low	No	No	Yes
TAU-Active	Antidepressants vs. antidepressants + CBT app^36^	Yes	High	No	No	Yes
mHealth Minimal Active	Psychoeducation control app vs. problem-solving therapy app^65^	Yes	High	Maybe	Yes	No
Dismantling Design	Mindfulness app with acceptance removed vs. mindfulness app^66^	Yes	High	Maybe	Maybe	No
Additive Design	Weight Watchers digital program vs. Weight Watchers digital program + just-in-time adaptive dietary lapse prevention smartphone app	Yes	High	Maybe	Maybe	No
mHealth Comparative Efficacy	Cognitive control app vs. problem-solving therapy app^65^	Yes	High	Maybe	Yes	No
non-mHealth Other Active	Weekend retreat alone vs. mindfulness and psychoeducation app^67^	Yes	High	Maybe	Yes	No
non-mHealth EBT	Clinician-guided online CBT vs. self-guided online CBT^68^	Yes	High	Maybe	Yes	No

*mHealth* mobile health, *EBT* evidence-based treatment (i.e., frontline intervention), *CBT* cognitive behavioral therapy.

### Decision tree

**Fig. 1:**
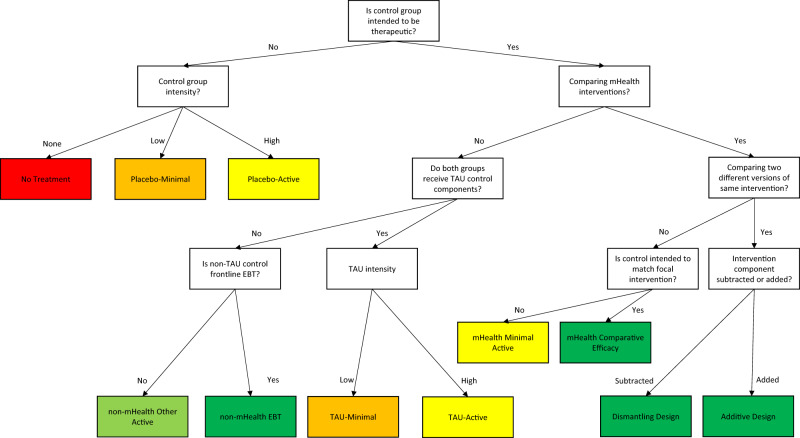
Eleven-category typology decision tree. mHealth mobile health, EBT evidence-based treatment (i.e., frontline intervention), TAU treatment-as-usual. Color coding reflects comparison strength ranging from low (Class V, red) to high (Class I, darker green). The choice of control condition type must ultimately be guided by the scientific question motivating a given study, which may be strongly influenced by a variety of factors including the stage of research (i.e., Class V comparisons may be very appropriate at early stages in the development of an intervention)^11,13,40^.

Figure 1 displays a decision tree with the 11 control condition types. Table 1 provides examples from the literature for each type. A first decision point is whether a control condition is intended to be therapeutic^28^. Non-therapeutic control conditions are further defined based on intensity: none (No Treatment), low (Placebo-Minimal), and high (Placebo-Active). The comparison strength increases as intensity increases.

There are a wide variety of control conditions that are, at least to some degree, intended to be therapeutic. Within therapeutic controls, the first distinction is whether the control condition is also an mHealth intervention. mHealth RCTs focused on isolating the effects of a particular intervention ingredient may use a Dismantling Design (i.e., therapeutic ingredient subtracted) or an Additive Design (i.e., therapeutic ingredient added)^34^. These designs provide a strong comparison for the specific ingredients being tested, although alone they cannot be used to evaluate the effects of an intervention as a whole. mHealth RCTs interested in comparing the efficacy of two or more mHealth interventions may use an mHealth Minimal Active control that is intended to be therapeutic but intended to be less potent than the focal intervention. The relative efficacy of two or more mHealth interventions can also be tested using a mHealth Comparative Efficacy control. mHealth Comparative Efficacy control conditions could be mHealth interventions with known efficacy (e.g., already FDA-approved)^3^, similar to comparisons conducted between novel pharmacotherapies and FDA-approved medications^35^.

mHealth RCTs often include non-mHealth intervention components. If both groups receive these elements, such control conditions are defined as TAU. However, TAU can vary in intensity, so this category is further graded into TAU-Minimal and TAU-Active. The determination of whether a control condition is TAU-Minimal or TAU-Active should be defined based on the context of standard care and the purpose of the focal intervention. For instance, for depression, TAU-Minimal could involve providing referrals to local clinicians with no additional follow-up^19^. An example of TAU-Active would be an active intervention received by all participants (e.g., antidepressants) to which mHealth is added as an adjunctive treatment^36^. For studying medication adherence among people living with HIV^21,37^, TAU-Minimal could be antiretroviral TAU (including the clinic’s standard care such as regular physician visits and relevant referrals). TAU-Active could include antiretroviral TAU along with an adherence-focused support group (i.e., an added element above and beyond standard care).

Some designs compare non-mHealth care with mHealth, and therefore do not provide non-mHealth care to the mHealth arm. These designs can provide a rigorous test of the relative efficacy of mHealth. We define a two-level distinction based on whether the non-mHealth control is a frontline evidence-based treatment (non-mHealth EBT Active), providing the highest comparison strength^38^, or another active control (non-mHealth Other Active) that is intended to be therapeutic but is not a frontline EBT.

### Scientific inferences

Table 2 describes the scientific inferences possible for each control group. There is no single control condition that is appropriate for every study at every stage of the research process^11,12,39^. Indeed, as discussed in previous guidelines^11,13^, clinical trialists are faced with balancing controlling threats to internal validity (where stronger comparisons provide more confidence that observed differences between intervention and control groups are due to the intervention itself) with statistical power (where stronger comparisons are expected to produce smaller between-group differences that require larger sample sizes for detection). In early stages of research^39,40^, No Treatment controls may be very appropriate in order to evaluate intervention effects beyond the passage of time and related confounds (e.g., regression to the mean)^41^. At later stages of research, stronger comparisons are warranted^13^. Placebo-Minimal controls for some non-specific factors (e.g., minimal amounts of time, attention, or expectancy) while a Placebo-Active can theoretically control for all non-specific factors, particularly when time and expectancy are matched. As noted, Dismantling Design and Additive Design controls are ideal for clarifying the effects of an intervention without a key ingredient or with a key ingredient added, respectively. Scientific questions related to comparisons with other mHealth interventions are best addressed using mHealth Minimal Active controls or mHealth Comparative Efficacy controls. These controls can establish the degree to which a focal intervention outperforms interventions expected to be of minimal strength (mHealth Minimal Active) or similar strength (mHealth Comparative Efficacy).

**Table 2:** Scientific inferences and comparison strength provided by each control condition.

Control condition	Scientific inference	Comparison strength
No Treatment	Intervention effects vs. passage of time/regression to the mean	Class V
Placebo-Minimal	Intervention effects beyond some non-specific factors (minimal time match, minimal expectancy)	Class IV
Placebo-Active	Intervention effects beyond non-specific factors (time match, expectancy)	Class III
TAU-Minimal	Intervention effects beyond minimal TAU	Class IV
TAU-Active	Intervention effects beyond active TAU, as adjunctive treatment	Class III
mHealth Minimal Active	Intervention effects relative to a minimal similar intervention	Class III
Dismantling Design	Intervention effects with component removed	Class I (for component)
Additive Design	Intervention effects with component added	Class I (for component)
mHealth Comparative Efficacy	Intervention effects relative to a similarly intensive mHealth intervention	Class I
non-mHealth Other Active	Intervention effects equivalent to established non-mHealth intervention	Class II
non-mHealth EBT	Intervention effects equivalent to frontline non-mHealth intervention	Class I

Comparison strength ranges from low (Class V) to high (Class I).

*mHealth* mobile health, *EBT* evidence-based treatment (i.e., frontline intervention).

Four control conditions can be used to evaluate the effects of mHealth in the context of non-mHealth interventions. Studies focused on evaluating the effect of augmenting treatment with mHealth can use a TAU-Minimal or TAU-Active control. These control conditions allow evaluation of the effects of mHealth as an adjunctive to another treatment. The final two control conditions address the degree to which a mHealth intervention performs on par with a non-mHealth intervention. Non-mHealth Other Active controls clarify whether mHealth interventions outperform non-EBT non-mHealth interventions while non-mHealth EBT Active controls clarify whether mHealth interventions outperform frontline, non-mHealth interventions.

### Comparison strength

The choice of control condition heavily impacts the comparison strength within a study^12^. Table 2 displays a five-tier scheme for grading the comparison strength provided by the 11 control conditions. No Treatment controls provide the lowest tier (Class V). Class IV comparison strength can be derived from either Placebo-Minimal or TAU-Minimal, as both of these control conditions are expected to be either non-therapeutic or minimally therapeutic and received by both groups. They provide only slightly stronger comparison than No Treatment controls. Class III comparison strength can be derived from Placebo-Active, TAU-Active, or mHealth Minimal Active controls. The Placebo-Active control provides a stronger comparison than the Placebo-Minimal in its ability to match on a larger number of non-specific factors (e.g., expectancy). Likewise, TAU-Active sets a higher bar for demonstrating the effects of mHealth than TAU-Minimal, with both arms receiving an active therapeutic intervention. mHealth Minimal Active provides evidence of effects beyond a limited mHealth intervention which, although stronger than comparisons with somewhat or entirely inert controls (Class IV and Class V, respectively), is only a moderately strong comparison given mHealth conditions are not matched.

The highest classes of comparison strength (Class II and I) require therapeutic control conditions that may be matched in intensity and non-specific factors including expectancy. These comparisons may be most appropriate for the later stages of intervention testing^39,40^. Non-EBT non-mHealth controls provide strong comparisons (Class II), but weaker comparisons than frontline, non-mHealth EBTs. Dismantling Design and Additive Design controls provide very strong comparisons (Class I), but only for the components being subtracted or added and not for the intervention as a whole. The highest comparison strength (Class I) for the mHealth intervention as a whole requires comparisons with either a matched mHealth Comparative Efficacy control or a non-mHealth EBT control.

### Alternative coding schemes

The 11-category scheme is designed to characterize important differences between control conditions in mHealth RCTs. Nonetheless, this scheme is detailed and there may be advantages to having simpler schemes (e.g., for meta-analysis). Here we propose four simplified schemes (Table 3).

**Table 3:** Alternative coding schemes.

Control condition	Five-category	Four-category	Two-category active vs. non-active	Two-category therapeutic vs. non-therapeutic
No Treatment	No Treatment	No Treatment	Passive	Not therapeutic
Placebo-Minimal	Placebo	Minimal Treatment	Active	Not therapeutic
Placebo-Active	Placebo	Non-Specific Active	Active	Not therapeutic
TAU-Minimal	TAU	No Treatment	Passive	Therapeutic
TAU-Active	TAU	Unclear	Unclear	Therapeutic
mHealth Minimal Active	mHealth Comparison	Minimal Treatment	Active	Therapeutic
Dismantling Design	mHealth Comparison	Specific Active	Active	Therapeutic
Additive Design	mHealth Comparison	Specific Active	Active	Therapeutic
mHealth Comparative Efficacy	mHealth Comparison	Specific Active	Active	Therapeutic
non-mHealth Other Active	non-mHealth Comparison	Specific Active	Active	Therapeutic
non-mHealth EBT	non-mHealth Comparison	Specific Active	Active	Therapeutic

*mHealth* mobile health, *EBT* evidence-based treatment (i.e., frontline intervention), *Non-specific active* active control not intended to be therapeutic, *Specific Active* active control intended to be therapeutic.

#### Five-category

A five-category scheme collapses across levels of placebo controls, TAU controls, mHealth controls, and non-mHealth controls. This scheme retains distinctions between controls intended to be therapeutic (TAU, mHealth controls, non-mHealth controls) and non-therapeutic (No Treatment, Placebo). Although this scheme can help describe the types of comparisons, it ignores variations in intensity and evidence base which may influence scientific inferences and effect sizes.

#### Four-category

This scheme also distinguishes between therapeutic and non-therapeutic controls, defined as non-specific and specific active controls^42,43^, while combining minimal treatment controls into a single group. The four categories include: No Treatment, Minimal Treatment (Placebo-Minimal and mHealth Minimal Active), Non-Specific Active (Placebo-Active), and Specific Active (Dismantling Design, Additive Design, mHealth Comparative Efficacy, non-mHealth Other Active, non-mHealth EBT). One control condition that is challenging to include in this scheme is TAU-Active. While it may be defensible to view a minimal amount of treatment received by both focal and controls arms as essentially canceling each other out (i.e., yielding a control condition more similar to a No Treatment control than an active control), this case is harder to make for TAU-Active controls that includes intensive, therapeutic interventions received by both groups (e.g., antidepressants)^36^.

#### Two-category: passive vs. active

A first two-category scheme involves differentiating between Passive vs. Active controls. In this scheme, Passive controls include No Treatment and TAU-Minimal. Active controls include the remaining conditions. A downside of this scheme is it ignores whether a control is intended to be therapeutic. From our perspective, this scheme is not ideal, although, of note, it is used within the meta-analytic literature^44^ and is arguably preferable to collapsing across all control conditions^24,45^.

#### Two-category: therapeutic vs. non-therapeutic

A second two-category scheme differentiates between control conditions intended to be therapeutic and not intended to be therapeutic^28^. We view this as the minimally viable distinction that should be made. However, it requires collapsing across a theoretically wide range of non-therapeutic (i.e., No Treatment, Placebo-Minimal, Placebo-Active) and therapeutic controls. This distinction is still likely superior to considering all control conditions the same.

## Open questions and future directions

The control condition coding schemes, ranging from simplistic two-category schemes to an 11-category scheme, are intended to highlight important differences between the variety of control conditions used in mHealth RCTs. At once, these schemes are all imperfect and are offered as an initial attempt to categorize the highly diverse mHealth literature. Here we discuss several open questions regarding categorizing control conditions in mHealth RCTs and highlight areas for future research.

### Fuzzy boundaries and edge cases

As an initial attempt, we have provided general guidance to distinguish between categories. However, it is currently unclear how several aspects should be handled, making the boundaries between categories fuzzy. One important issue is how precisely to determine whether focal and control interventions are matched on a particular dimension, especially in terms of intensity and expectancy. As noted, engagement with mHealth interventions can be highly variable^29,30^. Although focal and control interventions may both in theory be equally intensive, participants may not engage with them both equally. Similarly, expectancy may or may not actually be matched across conditions. Moreover, clinical trialists may reasonably disagree about how a particular control condition should be categorized. For example, one trial may describe ecological momentary assessment as “mood monitoring”^46,47^ and consider it a mHealth Minimal Active control while another considers it a Placebo-Active, Placebo-Minimal, or even No Treatment control. Arguably, it is incumbent upon mHealth trialists to demonstrate that control conditions intended to be intensity and/or expectancy matched are indeed matched. These data may inform how a control condition is described. For example, a placebo condition that fails to demonstrate adequate intensity and expectancy match may be better described as Placebo-Minimal or No Treatment than Placebo-Active. The boundary between TAU-Minimal and TAU-Active may often be fuzzy in contexts where TAU is heterogeneous (i.e., patients receive differing amounts and/or type of treatment).

Several other aspects of modern mHealth RCTs do not fit neatly into the 11-category scheme. Adaptive designs in which the treatment that is delivered changes over time (e.g., sequential multiple assignment randomized controlled trial [SMART])^48^ and factorial designs that evaluate multiple aspects of an mHealth intervention at once are challenging to categorize. Such studies collapse across various intervention elements in analyses to evaluate a range of comparisons within a single trial. For example, a SMART study may initially randomize participants to one of two app conditions (e.g., mHealth comparative efficacy control) but re-randomize participants who do not show symptom reductions after a set period of time to additional intervention components (e.g., coaching [non-mHealth Other Active]). Some analyses of SMART and factorial trials may involve a particular control condition type (e.g., analyses collapsing across conditions receiving a non-mHealth intervention component [non-mHealth Other Active]) while other analyses may involve another control condition type (e.g., analyses collapsing across conditions that did not receive a specific additional mHealth component [Additive Design]). Dismantling Designs, although categorized as intended to be therapeutic in our scheme, may have removed all theoretically therapeutic ingredients^34^ and therefore be better categorized as Placebo-Active.

The contribution of study-related human support in mHealth RCTs also makes categorization challenging. Studies commonly involve interaction between participants and study staff, although it can be hard to evaluate the extent and nature of this interaction from published RCTs^49^. Clinical trialists clearly reporting details regarding human support within RCTs and the degree to which such interactions may have been therapeutic can aid in categorization (e.g., a No Treatment control with ongoing, therapeutic human support may be better categorized as TAU-Minimal).

### Ethics

One issue not addressed by our scheme, but of paramount importance, is the ethical dimension of study design. Selecting a control condition has ethical implications around access to care, scientific rigor, and bias. Particularly in under-resourced settings and when working with vulnerable populations, No Treatment control conditions may be inappropriate as they may involve withholding interventions with known therapeutic value^50,51^. In contexts where trust is limited between community members and academic research teams, providing an active control may be necessary for building trust and completing recruitment and enrollment goals for an RCT. In these contexts, it may be more appropriate to provide at least a minimal degree of intervention (e.g., TAU-Minimal), for example links to publicly available online resources^52^. As a program of research matures (e.g., moves through the stages of intervention development)^11,39,40^, increasingly rigorous and theoretically therapeutic control conditions may be warranted, both for ethical (e.g., beneficence and non-maleficence)^53^ and scientific reasons (i.e., determining how a novel treatment compares with established treatments). As a general rule, clinical trials should only be run when there is the expectation that the treatment being studied may be beneficial (i.e., equipoise)^54^. The ethical use of digital health tools remains an active area of investigation, with frameworks emerging but none yet consistently adopted^55,56^.

### Application to meta-analysis

The proposed coding schemes are intended to be helpful for meta-analysts faced with categorizing the diverse range of control conditions used in mHealth RCTs. At a minimum, we hope this helps avoid considering control conditions to be a monolithic category^57^. Distinguishing between controls intended to be therapeutic and not intended to be therapeutic provides perhaps the simplest theoretically justified categorization. New meta-analyses sensitive to the impact of controls groups may provide more accurate effect size estimates and results of prior meta-analyses may need to be reexamined in light of these considerations.

### Limitations and future directions

As control conditions can dramatically impact effect sizes observed in RCTs of psychological interventions generally^58,59^ and mHealth RCTs specifically^44^, considering control conditions to be a single category is typically not defensible. The framework we propose here may help guide the selection of a control group, but it cannot definitively identify the ideal one for any particular study. Like all frameworks, its value must be proven in its utility. Thus, several future directions follow. A valuable next step would be the development and adoption of detailed control condition categorization guidelines that can be used by both clinical trialists and meta-analysts. It would be helpful to convene a Delphi panel of global experts for this purpose, which ideally would result in a checklist that can be included with mHealth RCTs to clarify and justify choice of control condition. Relatedly, it may be helpful to identify and/or develop specific measures that can be used to help delineate between similar control conditions (e.g., demonstrating intensity and expectancy match for Placebo-Active and mHealth Comparative Efficacy controls). It would be valuable to code the existing mHealth RCT literature using the schemes proposed here. A future meta-analysis could evaluate the degree to which between-group effect sizes vary across categories. In theory, between-group effect sizes should become smaller as the comparison strength increases from Class V to Class I. As consensus is reached regarding the categorization of control conditions in mHealth RCTs, this information can be used by bodies charged with regulating these technologies (e.g., FDA)^60^, researchers seeking to adequately power mHealth RCTs, industry stakeholders involved in investing and marketing, and ultimately patients and clinicians deciding when to use a given mHealth tool.
